# 

**Published:** 1885-11

**Authors:** 


					﻿N O V E M B E R , 1 8 8 5
(OLD SERIES
\ Vol. XXV

NEW SEfl^R?'
*	ii/__mv' q
The Atlanta
HBIGAL^U
^OURNA^^X

' W7. WESTMOREL AND. M:t)<
H.V.M.MILLER.M.D.LL.D.JK
JAMES.A.GRAY. M.D.^
PUBLISHED By james.p.harrison&co
<K	Atlanta,ga. AsM
SUBSCRIPTION: $2.50 A YEAR.
				

